# Confronting the Care Delivery Challenges Arising from Precision Medicine

**DOI:** 10.3389/fonc.2016.00106

**Published:** 2016-04-27

**Authors:** Elise C. Kohn, S. Percy Ivy

**Affiliations:** ^1^Cancer Therapy Evaluation Program, Division of Cancer Therapy and Diagnosis, National Cancer Institute, Rockville, MD, USA

**Keywords:** precision medicine, biomarkers, targets, genetic testing, opportunities, obstacles

## Abstract

Understanding the biology of cancer at the cellular and molecular levels, and the application of such knowledge to the patient, has opened new opportunities and uncovered new obstacles to quality cancer care delivery. Benefits include our ability to now understand that many, if not most, cancers are not one-size-fits-all. Cancers are a variety of diseases for which intervention may be very different. This approach is beginning to bear fruit in gynecologic cancers where we are investigating therapeutic optimization at a more focused level, that while not yet precision care, is perhaps much improved. Obstacles to quality care for patients come from many directions. These include incomplete understanding of the role of the mutant proteins in the cancers, the narrow spectrum of agents, broader mutational profiles in solid tumors, and sometimes overzealous application of the findings of genetic testing. This has been further compromised by the unbridled use of social media by all stakeholders in cancer care often without scientific qualification, where anecdote sometimes masquerades as a fact. The only current remedy is to wave the flag of caution, encourage all patients who undergo genetic testing, either germline or somatic, to do so with the oversight of genetic counselors and physician scientists knowledgeable in the pathways involved. This aspiration is accomplished with well-designed clinical trials that inform next steps in this complex and ever evolving process.

## Introduction

Cancer care delivery was once relatively simple due to the few available drugs, limited understanding of the complexity of the cancers, and less sophisticated diagnostic and therapeutic interventions. The exponential increase in knowledge brought about by microdissection of the genome, kinome, and other – omes, has yielded new classifications of cancers, classes of agents, different methods for dose determination, and increasing potential for personalization. This rapidly expanding knowledge creates the potential for diversity and inequality in cancer care.

Understanding the roles and limitations of these new resources narrows that treatment delivery gap. The harmonization of diagnostic approaches and expectations for each patient with a defined cancer histology and/or genomic subtype will assure the same care for all. Monitoring for treatment decisions should be consistent and driven by objective data, change in responsiveness, and/or toxicity parameters. Recognition of when the risk/benefit balance has shifted toward harm remains a critical element.

Optimization of cancer treatment in the molecular era is not always defined by the molecular make up of cancer. Characterization of the molecular biomarker utility across a type of cancer and then from the patient perspective needs to be coordinated. The charge to the molecular oncologist is to recognize when compelling data from high-level evidence identifies a molecular finding of therapeutic importance. In most cancers, this remains a goal. We have few validated biomarkers to guide us in women’s cancers and an abundance of molecular noise to dampen. Molecular testing is often done for reasons that the patients do not understand, and the testing costs thousands of dollars, frequently yielding little guidance. Determining and validating selective targets, target-drug pairing, and best patient practices will take carefully designed studies with well considered correlative science, requiring patients, time, and support. Currently, other than the use of germline *BRCA1/2* mutation testing and Lynch Syndrome testing, application of molecular diagnostics to the broad gynecologic cancer population is premature.

## Biomarkers: What, When, and Why

### Biomarkers, Definition?

A biomarker generally refers to a measured characteristic, which may be used as an indicator of some biological state or condition. Biomarkers may be developed to address multiple purposes related to patient diagnosis and selection, or drug and treatment effect. Molecular diagnostics can be translocations, such as *BCR-ABL* for chronic myelogenous leukemia, expression of mutant proteins or inappropriate protein expression, such as p53 in many solid tumors, or loss of expression as with E-cadherin loss in lobular breast cancer.

Molecular biomarkers can be used for therapeutic selection. Amplification of *HER2* is both a diagnostic and selective biomarker. It helps classify a type of breast cancer, and its presence determines targeted therapy selection. Identification of specific mutations in lung cancer, such as *EGFR* mutations, drives selection of the therapeutic classes of targeted agents. Alternatively, broad sequencing in a discovery mode can be used to determine targetable molecular events on a case-by-case basis. This is the hypothesis underlying the NCI MATCH study (NCT02465060) and other basket studies.

**Figure 1:**
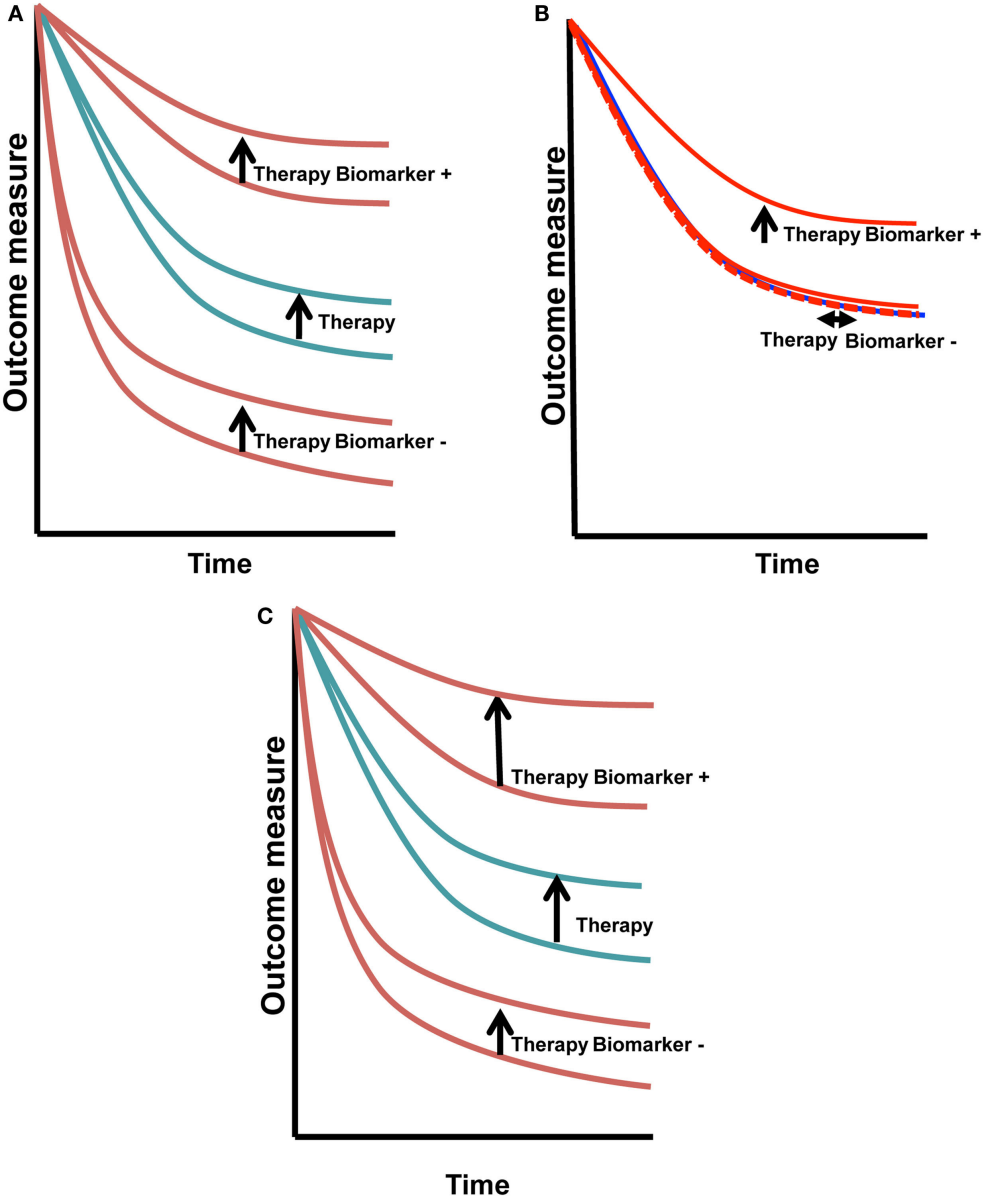
**Predictive vs. prognostic biomarkers**. Prognostic biomarkers **(A)** have similar change in outcome with therapy that is independent of the biomarker status. Predictive biomarkers **(B)** have a treatment/outcome interaction, seen in this example as improvement with treatment in biomarker^+^ cases (vertical arrow), with absence of change in the biomarker^−^ cases (horizontal arrow). Biomarkers that may be both prognostic and predictive **(C)** will shift the biomarker^+^ curve the same or greater if both the prognostic and predictive effects are positive. If one is positive and the other is negative, the outcome may cancel. This is a more complex situation to dissect.

Biomarkers also may be used as surrogates of clinical behavior, such as those readily measured in blood-like, CA-125 and PSA. These biomarkers may also be evaluated for prognostic and/or predictive potential. Prognostic biomarkers are those that dichotomize clinical outcomes, such as survival, in a therapy-agnostic fashion (Figure 1A). They are most often defined based upon correlative findings. Clinical biomarkers used commonly as prognostic directors in women’s cancers include stage, grade, age, lymphovascular space invasion, and number of positive lymph nodes (1).

Predictive biomarkers are most elusive and potentially most valuable (2, 3). They dichotomize outcomes in a therapy-specific fashion (Figure 1B). HER2, a diagnostic and selection biomarker, is both prognostic and predictive, shifting the full cohort outcome and biomarker positive patients in the upper set of curves (Figure 1C) (4, 5). HER-2^AMP^ breast cancer patients had a worse prognosis when given the same treatment as their HER-2 non-amplified counterparts. The introduction of HER-2 targeted therapy has changed that poor prognosis. Now, HER-2^AMP^ is a biomarker predictive of responsiveness to HER-2 targeted agents.

### Integral vs. Integrated Trial Biomarkers

Rigorous biomarker development is important. It requires qualification, optimization, and validation at levels of pre-analytic and analytical methods. Standard operating procedures for the collection and processing of patient-derived materials, pre-analytic methodologies, assure the collection of high-quality specimens. What samples, how they are taken, how and when they are processed, and the what/how/when of storage are critical pre-analytical variables (6). Quality control of reliability, reproducibility, variance, and cut-off determination are key analytical variables (7, 8).

Biomarkers that are required for the execution of a trial and/or the application of an agent are integral to the therapeutic direction. Integral biomarkers require the tightest pre-analytical and analytical standards, and if involved in patient care, must be done in appropriately certified laboratories. Integrated biomarkers are those that are included in clinical trials in hypothesis-directed objectives to be executed in a controlled, optimized fashion, to validate them for future integral application. Integral and integrated biomarkers use assay methodologies that are well past exploration and discovery and are moving toward anticipated use or standard of care. Understanding where a molecular biomarker is in development is critical to its proper application to the patient treatment setting.

### Fit-for-Purpose Biomarkers

The complexity of biomarker selection underscores the importance of using biomarkers that are fit-for-purpose (FFP). A FFP biomarker is defined by its intended use and by the biomarker assay method performance (2). The intended use or purpose of the biomarker or biomarker assay data is described in many ways, including pharmacokinetic, pharmacodynamic, diagnostic, exploratory, safety, enrollment, or companion diagnostic. A FFP biomarker is categorized as (a) integral, used for patient or treatment selection, (b) integrated, used to established treatment or disease state effects, or (c) exploratory, used descriptively or for screening for effects that are unestablished or poorly described. The stringency of the proposed assay method validation is defined and determined by the biomarker category, risk–benefit to the patient, and invasiveness. Biomarker assay method performance must be reliable and reproducible, and the assay must have well-defined performance characteristics. Performance metrics are qualitative and quantitative and include measures of sensitivity, specificity, accuracy, precision/robustness, stability, reference intervals/standards and cut-points (dynamic range), calibrators, range of quantification, dilutional linearity, sample re-analysis, interference, and normal signal distribution. Methodology performance evaluation is iterative and is updated throughout the life cycle of the biomarker development (9).

The circumstances under which biomarker testing is applied should be considered in the context of standard testing issues when planning a clinical trial. Each trial should have a biomarker prioritization plan. Parallel development of drugs and biomarkers is the key to rapid and purposeful progress. Many prognostic, but few predictive, biomarkers are under development. Such development is found in the translational literature and in clinical trial design where such questions are included.

## Optimizing Molecular Cancer Care Delivery for the Patient

### When Is It Precision Treatment or Scientific Experiment?

Optimal use of diagnostic and therapeutic resources requires understanding when there is potential for reliable data or when it is a shot in the dark. Few biomarkers have been validated in gynecologic cancers. They include the serum biomarkers for germ cell tumors (βHCG and αFP) (10), recurrent epithelial ovarian cancer (CA-125 and HE4) (11–14), endometrial cancer (HE4) (15), granulosa cell tumors (inhibin) (16), and the molecular markers of germline or somatic *BRCA1 or BRCA2* mutation (1, 17–19).

Driver molecular events have been identified and validated in some sarcomas and solid tumors. Drivers are molecular events that, based upon preclinical modeling and clinical testing, initiate, promote, and/or maintain malignancy in an obligatory fashion. Specific inhibition of driver(s) in patients results in dramatic clinical response. For example, the *bcr-abl* translocation results in constitutive activation of abl kinase and drives chronic myelogenous leukemia. Its inhibition with imatinib, dasatinib, and others is the gold standard example to define driver function (20). As impressive as these events are in preclinical models and in patients, to date, the only curative driver events have been identified in leukemias.

The most common drivers identified cause gain-of-function oncogenic behaviors, commonly by translocation, mutation, or occasionally by amplification. The regions in oncogenes that result in unfettered activation are few and are “hot spot” mutations for which focused screening can be done, or are identifiable breakpoints at translocations causing an activation event that can be readily identified. Similarly, oncogenic mutations, such as those seen in *c-kit* and *PDGFR* in gastrointestinal stromal tumors (21, 22) and translocations, such as the driving *ALK* translocation in non-small cell lung cancers (23), likewise, cause constitutive kinase or receptor activity. Inhibition of oncogenic signaling pathways by small molecule inhibitors results in impressive objective clinical effects (24, 25).

Inhibition of tumor suppressor genes is another mechanism through which carcinogenic behaviors are unmasked. Loss-of-function of tumor suppressor genes occurs with one of several events, such as mutational introduction of a stop codon that prevents transcription and translation of an active protein or by mutation that inactivates or alters function of the translated protein. These events are seen in p53 in ovarian and endometrial cancers. Unlike the hotspot mutational foci seen in oncogenic gain-of-function mutations or translocations, tumor suppressor gene mutations and rearrangements can and do occur all along the gene with “hot spots” that may identify population founder events. The more common gain-of-function p53 mutation is one where the mutation abrogates normal p53 checkpoint activity allowing cells to move through the cell cycle without stopping to repair the DNA damage. Protein is not lost and is seen as strong and broad staining of p53 by immunohistochemistry. The loss-of-function events, where p53 protein is lost, also have been identified in gynecologic cancers (26), and the early data suggest that there may be biological differences caused by the two mutational events (27). Yet, there are no validated specific or selective therapeutic opportunities related to p53 mutations. Thus, while serving a diagnostic and prognostic purpose, p53 has no targeted therapeutic direction or predictive value.

*BRCA*1 and *BRCA*2 are tumor suppressor genes. Homozygous genomic injury with resultant loss of both functional alleles has strong biologic effect in reduction of homologous recombination double-stranded DNA repair (28). Germline monoallelic loss predisposes to breast and ovarian cancers yielding a very high lifetime risk and has been used to trigger cancer prevention approaches. Recently, PARP inhibitors, a drug class within the broad category of DNA repair inhibitors, have been shown to be more active in women with germline loss (29). *BRCA* mutations have thus been validated as predictive biomarkers in this setting. *BRCA* mutation testing has been approved by the US FDA as a companion diagnostic for selection for treatment with olaparib; it is a predictive and selective biomarker approved as related specifically to treatment with the PARP inhibitor, olaparib, for women in fourth or later ovarian cancer recurrence. Despite inclusion in the EMA approval of olaparib, the role of somatic BRCA mutation remains unclear and has not been accepted by the US FDA. Clarification of homozygous mutation vs. single somatic mutation and issues of gene dosage should be addressed.

### When Is Molecular Testing Reasonable for Standard of Care Oncologic Intervention?

A strong family history alone does not predict accurately the full spectrum of women with *BRCA* mutation-associated ovarian cancer. Thus, NCCN and SGO recommend testing all women with high-grade serous ovarian cancer. This can have implications for the patient’s family if she is found to harbor a deleterious germline mutation, found in approximately 17% of the newly diagnosed high-grade serous ovarian cancer patients (30). Lack of mutation has not been shown to be of biologic value. Knowledge of *BRCA* status may have impact upon cancer care for investigational uses, as defined by clinical trial entry criteria, but in the US does not affect treatment opportunities until fourth treatment line. The effect on the patient and her family is also of importance and is addressed elsewhere in this Research Topic.

Risk panel testing, whole exome and genome testing, and testing of oncogene panels are done as “standard of care” in some centers and often requested in order to find something actionable. Panel testing is the examination of a series of potentially important risk genes, such as the BROCA panel defined by Swisher and colleagues (31, 32). It includes the Lynch Syndrome genes and other genes with low frequency, but deleterious germline mutations, including *PALB2*, *RAD51*c, and *RAD51d*. Mutations in these genes may be linked to the risk of ovarian and other cancers, a prognostic event, but there is no validated predictive function (31). There are no data that exome or whole genome sequencing is medically useful or cost-effective for gynecologic cancer patients. Too often, this testing is presented to or by the patient as an expectation, related to receipt of care. The facts and foibles are not presented in depth, and often no or minimal informed consent is done, since many of these are commercially available. This includes not fully informing the patient about the financial implication and the support or lack thereof by their insurance coverage. The number of truly actionable events, where there are validated clinical outcomes linked to genomic findings, are exceptionally rare in gynecologic cancers and do not inform patient care. Such testing should be done in the context of a clinical trial, such as the NCI MATCH (NCT02465060).

### Opportunities and Obstacles

Molecular characterization of gynecologic and other cancers created a great opportunity for learning about the behavior of the cancer, its heterogeneity, how subclones outgrow during treatment, and to identify therapeutic opportunities (Table 1). These prospects may have little benefit to the individual patient but in aggregate may provide key information that, when mined, can yield important new insights. This was demonstrated by the remarkable progress occurring after broader characterization of *BRCA* mutation carriers. Those advances resulted in identification of the precursor fallopian tube lesion for ovarian cancer, an understanding of the importance of different mechanisms of DNA repair, and the advancement of several new classes of DNA repair inhibitory agents.

**Table 1:** **Obstacles and opportunities of molecular testing in gynecologic cancers**.

Opportunities	Obstacles
Advance understanding of cancer(s)	Intrinsic cancer elements
• Identify novel drivers and facilitators	• Unclear functional status of mutation
• Examine heterogeneity	• Heterogeneity
• Dissect cause of molecular events	• Tumor–microenvironment interactions
	• Molecular divergence
	• Activation of secondary pathways
Knowledge on a per-patient basis for therapeutic selection	Selection approaches may miss optimal personal opportunities
Translate science to therapeutic opportunities	Mechanisms of resistance and risk of negating effects of subsequent targeted agents
Drive novel trial designs and statistical models	Cost: patient time (from work, travel, etc), assay costs, and physician and counseling costs
	Low clinical trial participation

The further understanding of molecular aspects of cancer has resulted in novel trial designs and statistical models. Trial designs, categorizing therapy based upon common molecular events, such as NCI MATCH (NCT02465060), are examining tissue for molecular events. It then seeks to match the molecular event to a drug that may target the molecular event. This study recognizes that the role of the molecular event in a given cancer is unknown. This is a direction to refer women with more rare ovarian cancers for which trials are not available and phase 1 options may be limited.

Several studies have been published with similar target-matching approaches. The SHIVA investigators found that the use of molecularly targeted agents outside their indications does not improve outcome over physician’s choice, underscoring the requirement for understanding the biology and selection opportunities within cancer/drug pairing (33). Schwaederle and colleagues (34) showed clinical benefits in the arm in which patients were matched to therapeutics by molecular targets over the arm with standard of care treatment. However, it did not present the cancer breakdown of the participating patients, preventing readers from determining if the positive results may have been driven by an overabundance of cancers with proven targets, such as non-small cell lung cancer subsets. Another study evaluated the role of the use of selection biomarkers in clinical trials of FDA-approved agents (35). This study showed improvement with the application of selection biomarkers where there was a validated biomarker for the targeted agent. These conflicting observations raise caution to the blanket application of costly sequencing. An alternative is the examination of exceptional responders (36). Finding mutational events and not being able to determine the role of those molecular changes can result in misdirection of therapy and potentially harm the patient clinically and economically, and importantly, can dash their hope by lack of success.

The explosion in understanding about the molecular basis of cancer, especially in women’s cancers, and in new agents, provides an important opportunity for patient education. The physician can frame the progress in genomics against the background of new agent availability. This can lead to a more informed joint decision as to whether referral to a screening/treating trial, such as MATCH, or for testing is appropriate for the patient at her point in her disease.

Heterogeneity provides some insight into the paucity of cures with targeted therapies. A great obstacle to application of personalized molecular medicine at this time appears to be cancers themselves. Solid tumors have some, or many, molecular events, often of uncertain importance, making targeted therapy more difficult to select. Discerning driver mutations from facilitating mutations from passenger mutations with no biologic consequence remains empiric. It is often further complicated by secondary mutations in many cases. *PI3K* mutations are a case in point. Almost all epithelial solid tumors have some form of PI3K pathway mutation or dysfunction (37, 38). PI3K inhibitors have been uniformly disappointing in solid tumors, while being approved for use in lymphomas where there are no PI3K mutations, but there is strong pathway activation. The next obstacle, a consequence of the molecular variability seen in most solid tumors, is intratumoral heterogeneity. Sequencing over disparate geographic areas has demonstrated intratumor molecular heterogeneity and allowed determination of temporal and spatial clonality (39, 40). It has demonstrated that divergence can be an early event.

## Conclusion

The promise of personalized molecular medicine has been long in being recognized, although clear progress is evident. Gynecologic cancers are complex, and focused attention to their genomics, biology, and local tumor microenvironment has yielded important clues to new therapeutic directions. While few clear drivers have been identified, selection parameters, including DNA repair dysfunction, are seen with the role of germline BRCA mutations and Lynch syndrome biology. The major opportunity and challenge ahead is to develop and validate the tools necessary to optimize the application of biomarkers and targeted agents to rapidly and efficiently improve cancer care delivery to women with gynecologic cancers.

## Author Contributions

EK and SI: design, writing, and editing of the manuscript.

## Conflict of Interest Statement

The authors declare that the research was conducted in the absence of any commercial or financial relationships that could be construed as a potential conflict of interest. The handling editor ST declared a shared affiliation, though no other collaboration with one of the authors, EK, and states that the process nevertheless met the standards of a fair and objective review.
